# Diagnostic Accuracy of Fluorine-18-Fluorodeoxyglucose Positron Emission Tomography in the Evaluation of the Primary Tumor in Patients with Cholangiocarcinoma: A Meta-Analysis

**DOI:** 10.1155/2014/247693

**Published:** 2014-05-13

**Authors:** Salvatore Annunziata, Carmelo Caldarella, Daniele Antonio Pizzuto, Federica Galiandro, Ramin Sadeghi, Luca Giovanella, Giorgio Treglia

**Affiliations:** ^1^Institute of Nuclear Medicine, Catholic University of the Sacred Heart, Largo Agostino Gemelli 8, 00168 Rome, Italy; ^2^Postgraduate School of General Surgery, Catholic University of the Sacred Heart, Largo Agostino Gemelli 8, 00168 Rome, Italy; ^3^Nuclear Medicine Research Center, Mashhad University of Medical Sciences, Daneshgah Avenue, Mashhad 9179817317, Iran; ^4^Department of Nuclear Medicine and PET/CT Center, Oncology Institute of Southern Switzerland, Via Ospedale 12, 6500 Bellinzona, Switzerland

## Abstract

*Objective.* To meta-analyze published data about the diagnostic accuracy of fluorine-18-fluorodeoxyglucose (^18^F-FDG) positron emission tomography (PET) or PET/computed tomography (PET/CT) for primary tumor evaluation in patients with cholangiocarcinoma (CCa). * Methods. * A comprehensive literature search of studies published through December 31, 2013, was performed. Pooled sensitivity and specificity were calculated on a per patient based analysis. Subgroup analyses considering the device used (PET versus PET/CT) and the localization of the primary tumor (intrahepatic cholangiocarcinoma (IH-CCa), extrahepatic cholangiocarcinoma (EH-CCa), and hilar cholangiocarcinoma (H-CCa)) were carried out. * Results. *Twenty-three studies including 1232 patients were included in the meta-analysis. Pooled sensitivity and specificity of ^18^F-FDG-PET or PET/CT were 81% and 82%, respectively. Pooled sensitivity and specificity, respectively, were 80% and 89% for PET, 82% and 75% for PET/CT, 95% and 83% for IH-CCa, 84% and 95% for H-CCa, and 76% and 74% for EH-CCa. * Conclusions.* ^18^F-FDG-PET and PET/CT were demonstrated to be accurate diagnostic imaging methods for primary tumor evaluation in patients with CCa. These tools have a better diagnostic accuracy in patients with IH-CCa than in patients with EH-CCa. Further studies are needed to evaluate the accuracy of ^18^F-FDG-PET or PET/CT in patients with H-CCa.

## 1. Introduction

Cholangiocarcinoma (CCa) is a malignant tumor arising from the epithelium of the bile ducts and is usually classified by anatomical and clinical criteria into intrahepatic cholangiocarcinoma (IH-CCa), hilar cholangiocarcinoma (H-CCa), and extrahepatic cholangiocarcinoma (EH-CCa) [1]. CCa has a poor prognosis and surgical resection with appropriate lymph node dissection is advocated as the curative approach in some patients [2]. Consequently, accurate evaluation and staging are critical to provide indication to surgery and to avoid unnecessary surgical interventions [3].

Several diagnostic tools have been used in this setting, including ultrasonography (US), computed tomography (CT), magnetic resonance (MR), endoscopic retrograde cholangiopancreatography (ERCP), and percutaneous transhepatic cholangiography (PTC).

Fluorine-18-fluorodeoxyglucose (^18^F-FDG) positron emission tomography (PET) and PET/CT have been proposed as noninvasive imaging methods to assess the disease extent in cancer patients [4]. Since ^18^F-FDG is a glucose analogue, this radiopharmaceutical may be very useful in detecting malignant lesions which usually present high glucose metabolism. Hybrid PET/CT device allows enhanced detection and characterization of neoplastic lesions, by combining the functional data obtained by PET with morphological data obtained by CT [4].

Several studies have assessed the diagnostic accuracy of ^18^F-FDG-PET or PET/CT in the evaluation of primary tumor in patients with CCa, reporting different values of sensitivity and specificity. The purpose of our study is to meta-analyze published data on the diagnostic accuracy of ^18^F-FDG-PET or PET/CT in the evaluation of primary tumor in patients with CCa, in order to provide more evidence-based data and to address further studies in this setting.

## 2. Materials and Methods

### 2.1. Search Strategy

A comprehensive computer literature search of PubMed/MEDLINE and Embase databases was carried out to find relevant published articles concerning the evaluation of primary tumor in patients with CCa. We used a search algorithm based on a combination of terms (“PET” or “positron emission tomography”) and (“cholangiocarcinoma” or “cholangiocellular” or “cholangio∗” or “biliar” or “biliary” or “bile” or “Klatskin”). Only articles in English language were considered. The search was performed from inception to December 31, 2013. To expand our search, references of the retrieved articles were also screened for additional studies.

### 2.2. Study Selection

Studies or subsets in studies investigating the role of ^18^F-FDG-PET or PET/CT in the evaluation of primary CCa were eligible for inclusion. Case reports, small case series, review articles, letters, editorials, and conference proceedings were excluded. The following inclusion criteria were applied to select studies for this meta-analysis:

1. original studies in which ^18^F-FDG-PET or PET/CT were performed in patients with CCa or suspicious CCa;
2. a sample size of at least ten patients with CCa or suspicious CCa;
3. sufficient data to reassess sensitivity and specificity of ^18^F-FDG-PET or PET/CT in detecting the primary tumor in patients with CCa;
4. no data overlap.

Three researchers (SA, DAP, and CC) independently reviewed titles and abstracts of the retrieved articles, applying the above-mentioned selection criteria. Articles were rejected if they were clearly ineligible. The same three researchers then independently evaluated the full-text version of the included articles to determine their eligibility for inclusion.

### 2.3. Data Extraction

Information about basic study (authors, year of publication, and country of origin), study design (prospective or retrospective), patients' characteristics (number of patients with biliary ducts lesions performing ^18^F-FDG-PET or PET/CT, mean age, and gender), and technical aspects (injected activity of ^18^F-FDG and time between injection and image acquisition) was collected.

Each study was analyzed to retrieve the number of true-positive (TP), true-negative (TN), false-positive (FP), and false-negative (FN) findings of ^18^F-FDG-PET or PET/CT in patients with CCa or suspicious CCa, according to the reference standard. Only studies providing such complete information were finally included in the meta-analysis.

### 2.4. Quality Assessment

The 2011 Oxford Center for Evidence-Based Medicine checklist for diagnostic studies was used for quality assessment of the included studies. This checklist has 5 major parts as follows: representative spectrum of the patients, consecutive patient recruitment, ascertainment of the gold standard regardless of the index test results, independent blind comparison between the gold standard and index test results, and enough explanation of the test to permit replication.

### 2.5. Statistical Analysis

Sensitivity and specificity of ^18^F-FDG-PET and PET/CT in the evaluation of primary CCa were obtained from the individual studies, on a per patient-based analysis. We considered as positive a biliary ducts lesion with increased uptake of ^18^F-FDG, according to the criteria reported by the different authors. When a positive lesion was histologically confirmed as malignant, this was considered a TP lesion, whereas a histologically confirmed benign lesion was considered as a FP lesion. We considered as negative a lesion with no uptake of ^18^F-FDG: when the lesion was histologically confirmed as malignant, this was considered as FN lesion, whereas a histologically confirmed benign lesion was considered as a TN lesion.

Sensitivity was determined according to the following formula: TP/(TP + FN); specificity was determined according to this following formula: TN/(TN + FP). Statistical pooling of the data was performed by means of a random effects model. Pooled data are presented with 95% confidence intervals (95% CI). Heterogeneity between studies was assessed by an *I* ^2^ index. A summary receiving operator characteristics (ROC) curve was obtained for selected studies and area under the curve (AUC) was calculated to assess the overall accuracy of ^18^F-FDG-PET and PET/CT.

Subsequently, subgroup analyses were also performed, calculating the pooled sensitivity and specificity of ^18^F-FDG-PET and PET/CT in three different groups of primary CCa (IH-CCa, EH-CCa, and H-CCa) and in two groups based on the different device used (PET or PET/CT).

For publication bias evaluation, funnel plots, Egger's regression intercept, and Duval and Tweedie's method were used [5].

Statistical analyses were performed using Meta-DiSc statistical software version 1.4.

## 3. Results

### 3.1. Literature Search

**Figure 1:**
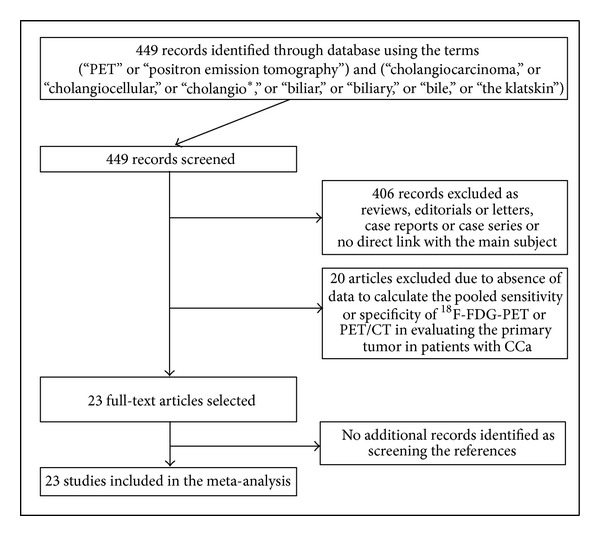
Plot of the literature search.

The comprehensive computer literature search from PubMed/MEDLINE and Embase databases revealed 449 articles. Reviewing titles and abstracts, 406 records were excluded as reviews, editorials or letters, case reports or case series, or no direct link with the main subject. Twenty articles were excluded due to absence of data to reassess the pooled sensitivity or specificity of ^18^F-FDG-PET or PET/CT in evaluating the primary tumor in patients with CCa or suspicious CCa. Finally, 23 articles including 1232 patients were selected and were eligible for the meta-analysis [1–3, 6–25]; no additional studies were found screening the references of these articles (Figure 1). The characteristics of the included studies are presented in Tables 1, 2, 3 and 4.

### 3.2. Qualitative Analysis (Systematic Review)

Using the database search, 23 original articles written over the past 12 years were selected [1–3, 6–25]. About the study design, 7 of these studies were prospective [6, 7, 13, 14, 17–19] and 12 were retrospective [1–3, 9, 12, 15, 16, 20–22, 24, 25] and in 4 articles this information was not provided [8, 10, 11, 23].

Ten studies used hybrid PET/CT [1–3, 11, 13, 18, 19, 21–23], whereas thirteen studies used PET only [6–10, 12, 14–17, 20, 24, 25]. Heterogeneous technical aspects between the included studies were found (Table 2). PET image analysis was performed by using qualitative criteria (visual analysis) in all the included studies [1–3, 6–25] and adjunctive semiquantitative criteria (based on the calculation of the standardized uptake value (SUV)) in 19 articles [1–3, 6–9, 11, 13, 15, 16, 18–25]. One study used quantitative criteria (based on blood sampling and the Gjedde-Patlak linearization procedure) [14].

The reference standard used to validate the ^18^F-FDG-PET or PET/CT findings in the included studies was quite different (Table 4). The results of the quality assessment of the studies included in this systematic review, according to the 2011 Oxford Center for Evidence-Based Medicine checklist for diagnostic studies, are shown in Table 4.

### 3.3. Quantitative Analysis (Meta-Analysis)

**Figure 2:**
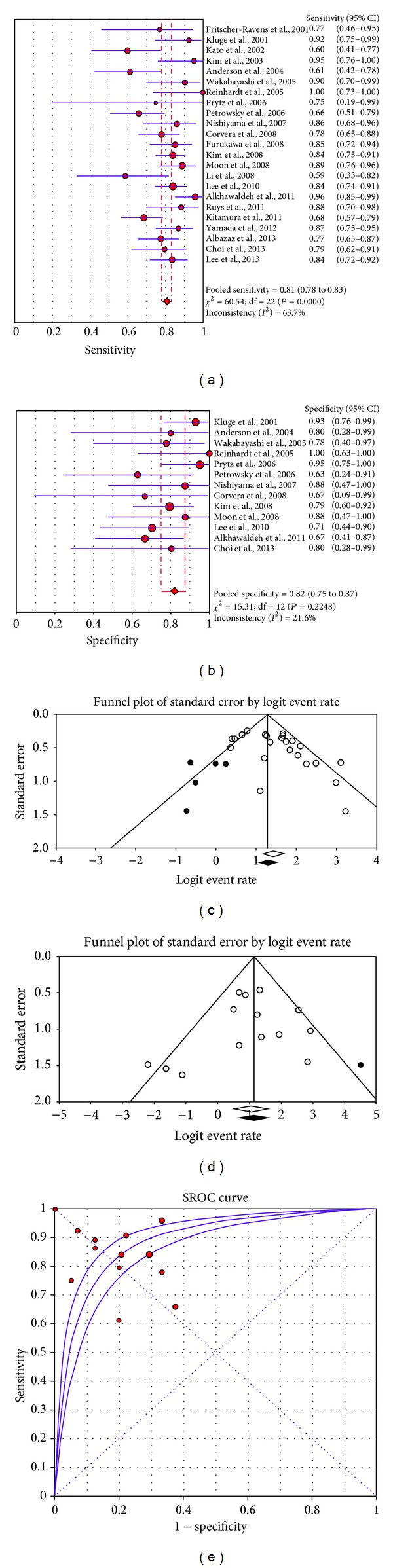
Plots of pooled sensitivity (a) and specificity (b), publication bias analysis for sensitivity (c) and specificity (d), and summary ROC curve (e) of ^18^F-FDG-PET or PET/CT in primary cholangiocarcinoma.

**Figure 3:**
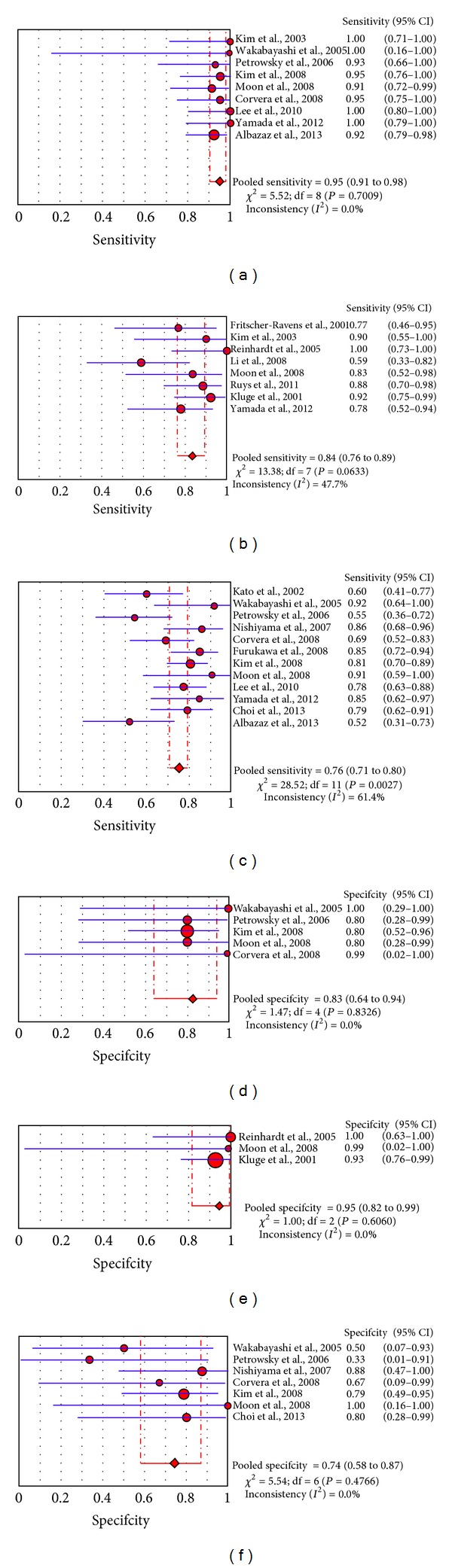
Plots of pooled sensitivity and specificity of ^18^F-FDG-PET or PET/CT in primary intrahepatic cholangiocarcinoma ((a), (d)), hilar cholangiocarcinoma ((b), (e)), and extrahepatic cholangiocarcinoma ((c), (f)).

**Figure 4:**
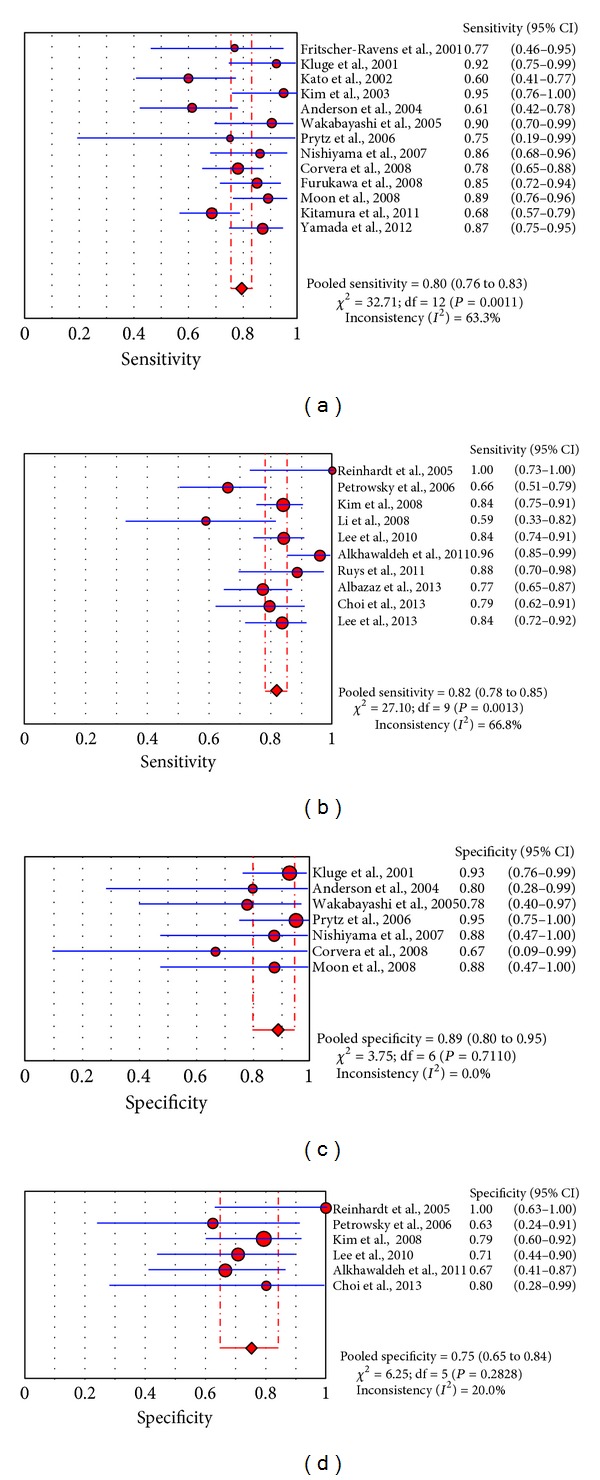
Plots of pooled sensitivity and specificity of ^18^F-FDG-PET ((a), (c)) or PET/CT ((b), (d)) in primary cholangiocarcinoma.

The diagnostic accuracy values of ^18^F-FDG-PET and PET/CT in the 23 studies included in the meta-analysis are presented in Figures 2–4. All the 23 studies had sufficient data to calculate the pooled sensitivity [1–3, 6–25], whereas only 13 studies [1, 7, 10–16, 18, 20–22] provided information about TN and FP lesions, thus allowing assessment of pooled specificity.

Sensitivity and specificity values of ^18^F-FDG-PET or PET/CT on a per patient-based analysis ranged from 59 to 100% and from 63 to 100%, with pooled estimates of 81% (95% CI: 78–83%) and 82% (95% CI: 75–87%), respectively. The area under the summary ROC curve was 0.89. The included studies showed statistical heterogeneity in their estimate of sensitivity (*I* ^2^: 63.7%).

Egger's regression intercepts for sensitivity and specificity pooling were 1.9 (95% CI: 0.3 to 3.5, *P* = 0.02) and −0.7 (95% CI: −2.4 to 0.9, *P* = 0.35), respectively. Applying Duval and Tweedie's method, the funnel plot of sensitivity and specificity reached symmetry and the adjusted sensitivity and specificity decreased 2.4% and increased 1.8%, respectively (Figure 2).

To reduce the heterogeneity, subgroup analyses considering the different device used (PET or PET/CT) were performed (Figure 4). In studies in which ^18^F-FDG-PET was used, values of sensitivity (thirteen eligible studies) and specificity (seven eligible studies) on a per patient-based analysis ranged from 60 to 95% and from 67 to 95%, respectively, with pooled estimates of 80% (95% CI: 76–83%) and 89% (95% CI: 80–95%), respectively. Statistical heterogeneity was found only in their estimate of sensitivity (*I* ^2^: 63%). The area under the ROC curve was 0.92.

In studies in which hybrid ^18^F-FDG-PET/CT was used, values of sensitivity (ten eligible studies) and specificity (six eligible studies) on a per patient-based analysis ranged from 59 to 100% and from 63 to 100%, respectively, with pooled estimates of 82% (95% CI: 78–85%) and 75% (95% CI: 65–84%), respectively. Statistical heterogeneity was found only in their estimate of sensitivity (*I* ^2^: 67%). The area under the ROC curve was 0.81.

Finally, subgroup analyses considering different anatomic sites of CCa (IH-CCa, EH-CCa, and H-CCa) were carried out (Figure 3). In patients with IH-CCa, values of sensitivity (nine eligible studies) and specificity (five eligible studies) on a per patient-based analysis ranged from 91 to 100% and from 80 to 100%, respectively, with pooled estimates of 95% (95% CI: 91–98%) and 83% (95% CI: 64–94%), respectively. No statistical heterogeneity was found, among the included studies, in both the estimate of sensitivity and the estimate of specificity (*I* ^2^: 0%). The area under the ROC curve was 0.95.

In patients with EH-CCA, values of sensitivity (twelve eligible studies) and specificity (seven eligible studies) on a per patient-based analysis ranged from 52 to 92% and from 33 to 100%, respectively, with pooled estimates of 76% (95% CI: 71–80%) and 74% (95% CI: 58–87%), respectively. Statistical heterogeneity was found only in their estimate of sensitivity (*I* ^2^: 61%). The area under the ROC curve was 0.82.

In patients with H-CCA, values of sensitivity (eight eligible studies) and specificity (three eligible studies) on a per patient-based analysis ranged from 59 to 100% and from 93 to 100%, respectively, with pooled estimates of 84% (95% CI: 76–89%) and 95% (95% CI: 82–99%), respectively. No significant statistical heterogeneity was found in their estimate of sensitivity (*I* ^2^: 48%) and specificity (*I* ^2^: 0%). The area under the ROC curve was 0.98.

## 4. Discussion

To the best of our knowledge, this meta-analysis is the first to evaluate the diagnostic accuracy of ^18^F-FDG-PET or PET/CT in the evaluation of primary tumor in patients with CCa [26]. Several studies have used ^18^F-FDG-PET or PET/CT in this setting reporting different values of sensitivity and specificity. However, many of these studies have limited power, analyzing only relatively small numbers of patients. In order to derive more robust estimates of the diagnostic accuracy of ^18^F-FDG-PET or PET/CT in this setting we pooled published studies. A systematic review process was adopted in ascertaining studies, thereby avoiding selection bias.

Pooled results of our meta-analysis indicate that ^18^F-FDG-PET or PET/CT have a good sensitivity (81%) and specificity (82%) in the evaluation of primary tumor in patients with CCa. Furthermore, the value of the AUC (0.89) demonstrates that ^18^F-FDG-PET or PET/CT are accurate diagnostic methods in this setting. Considering patients with all anatomical localizations of primary CCa, independently of the device used (PET or PET/CT), significant heterogeneity between the studies in their estimate of sensitivity was found (*I* ^2^: 63.7%). In order to reduce possible source of heterogeneity, subgroup analyses considering different device used (PET or PET/CT) and patients with different anatomical localizations (IH-, H-, and EH-CCa) were performed.

These subgroup analyses provide differences in the diagnostic accuracy data for various anatomical localizations. ^18^F-FDG-PET and PET/CT seem to be more sensitive and specific in the evaluation of primary tumor in patients with IH-CCA than in patients with H-CCA and EH-CCA.

In particular ^18^F-FDG-PET and PET/CT have a moderate diagnostic accuracy in evaluating primary EH-CCa (sensitivity of 76% and specificity of 74%). In this setting, sensitivity and specificity of ^18^F-FDG-PET and PET/CT may be affected by FN (due to the confounding anatomical localization of extrahepatic bile ducts) and FP (due to inflammation of extrahepatic bile ducts). Larger use of hybrid PET/CT and, consequently, further studies about the role of PET/CT in evaluation of primary tumour in patients with EH-CCA may improve these results.

Conversely, the diagnostic accuracy of ^18^F-FDG-PET and PET/CT in primary IH-CCA (sensitivity of 95% and specificity of 83%) seems to be better than in the other anatomical localizations of primary CCa. Possible explanations are the easier individuation of illness in the liver parenchyma and the small number of FP cases (intrahepatic noncancerous disease positive with ^18^F-FDG-PET). Further studies are needed to evaluate if different histological types of IH-CCA (nodular or mass-forming type, infiltrating type, and intraluminal type) could cause different diagnostic accuracy of ^18^F-FDG-PET and PET/CT in this setting.

Finally, the diagnostic accuracy of ^18^F-FDG-PET and PET/CT in evaluating primary H-CCa is good (sensitivity of 84% and specificity of 95%). Nevertheless, we cannot exclude that the low number of the included studies in this subgroup analysis may have influenced the results. FP findings (due to the presence of ^18^F-FDG-avid lymph nodes in the hepatic hilum) and FN results (due to the difficult anatomical localization of the hepatic hilum) should be considered. More studies are needed to further evaluate sensitivity and specificity of ^18^F-FDG-PET and PET/CT in primary H-CCa.

However, performing these subgroup analyses has been useful in demonstrating that the anatomical localization of primary tumor (IH-CCa, EH-CCa, or H-CCA) is a source of heterogeneity among the studies. In fact, no significant heterogeneity was found in the subgroup analyses performed, except in the calculation of pooled sensitivity of ^18^F-FDG-PET or PET/CT in primary EH-CCA.

Pooled sensitivity is similar in the subgroup analyses regarding different device used (80% for PET and 82% for PET/CT, resp.). Nevertheless, heterogeneity was found in these groups, in particular for the calculation of pooled sensitivity, suggesting that, beyond the device used, other factors (such as the anatomical localization of the primary CCa) seem to be a stronger source of heterogeneity. PET alone seems to be more specific than PET/CT (89% and 75%, resp.). A possible explanation of these surprising findings could be the higher number of patients with primary EH-CCa included in the studies which performed PET/CT compared to those which performed PET only.

Finally, regarding the diagnostic workup of patients with CCa, ^18^F-FDG-PET and PET/CT may have little diagnostic advantage over traditional imaging modalities in detecting the primary CCA [3]. ^18^F-FDG-PET and PET/CT can be complementary to CT and MR in the diagnosing and staging of CCA [20]. Since ^18^F-FDG-PET imaging is a whole-body scanning technique, it allows detection of unsuspected metastatic lymph nodes or distant spread that may lead to major changes in the surgical management of patients with biliary tract cancer [25]. Nevertheless, the diagnostic performance of ^18^FDG-PET or PET/CT in detecting metastatic lymph nodes or distant spread was not object of our analysis.

This study has several limitations. Different anatomical classifications of CCa were used by several studies. For example, it is likely that some H-CCa were classified as EH-CCa by some studies. Other possible limitations of our meta-analysis could be the heterogeneity between the included studies (nevertheless subgroup analyses were performed to reduce the heterogeneity) and the possible publication bias. We assessed publication bias in our meta-analysis using qualitative and quantitative methods (Egger's regression and Duval and Tweedie's method). Funnel plots showed the importance of possible publication bias in particular for the estimation of pooled sensitivity (Figure 2).

Overall, ^18^F-FDG-PET and PET/CT were demonstrated to be accurate noninvasive tools in the evaluation of primary tumors in patients with CCa. Furthermore, more studies in patients with H-CCa and cost-effectiveness analyses of the role of ^18^F-FDG-PET or PET/CT in this setting are needed.

## 5. Conclusions

^18^F-FDG-PET and PET/CT were demonstrated to be accurate diagnostic imaging methods in the evaluation of primary tumors in patients with CCa. These tools seem to have a better diagnostic accuracy in the evaluation of primary IH-CCa compared to EH-CCa. Further studies are needed to evaluate the accuracy of ^18^F-FDG-PET and PET/CT in assessing primary H-CCa.

## Figures and Tables

**Table 1:** Basic characteristics of the included studies.

Authors	Year	Country	Study design	Patients performing ^18^F-FDG-PET or PET/CT	Mean age (years)	Gender (% male)	Site of primary tumour
Fritscher-Ravens et al. [6]	2001	Germany	Prospective	15	58	60%	15 H-CCa

Kluge et al. [7]	2001	Germany	Prospective	54	NR	54%	NR

Kato et al. [8]	2002	Japan	NR	30	68	70%	30 EH-CCa

Kim et al. [9]	2003	Korea	Retrospective	21	57	52%	10 H -CCa and 11 IH-CCa

Anderson et al. [10]	2004	USA	NR	36	63	55%	NR

Reinhardt et al. [11]	2005	Germany	NR	20	63	50%	20 H-CCa

Wakabayashi et al. [12]	2005	Japan	Retrospective	30	71	50%	5 IH-CCa and 25 EH-CCa

Petrowsky et al. [13]	2006	Switzerland	Prospective	61	64	51%	14 IH-CCa and 33 EH-CCa

Prytz et al. [14]	2006	Sweden	Prospective	24	39	83%	NR

Nishiyama et al. [15]	2007	Japan	Retrospective	37	70	59%	29 EH-CCa

Corvera et al. [16]	2008	USA	Retrospective	126	62	52%	41 EH-CCa and 21 IH-CCa

Furukawa et al. [17]	2008	Japan	Prospective	72	69	57%	64 EH-CCa

Kim et al. [18]	2008	Korea	Prospective	123	60	65%	36 IH-CCa and 87 EH-CCa

Li et al. [19]	2008	Germany	Prospective	17	62	65%	17 H-CCa

Moon et al. [20]	2008	Korea	Retrospective	54	59	63%	23 IH-CCa, 12 H-CCa, and 11 EH-CCa

Lee et al. [21]	2010	Korea	Retrospective	99	67	59%	17 IH-CCa and 49 EH-CCa

Alkhawaldeh et al. [22]	2011	Germany	Retrospective	65	63	60%	34 H-CCa and 23 IH-CCa

Kitamura et al. [23]	2011	Japan	NR	73	66	63%	45 H-CCa and 28 EH-CCa

Ruys et al. [24]	2011	Netherlands	Retrospective	30	62	47%	26 H-CCa

Yamada et al. [25]	2012	Japan	Retrospective	73	68	63%	16 IH-CCa, 18 H-CCa, and 20 EH-CCa

Albazaz et al. [3]	2013	UK	Retrospective	81	65	41%	47 IH-CCa and 34 EH-CCa

Choi et al. [1]	2013	Korea	Retrospective	39	64	72%	34 EH-CCa

Lee et al. [2]	2013	Korea	Retrospective	52	69	53%	23 EH-CCa, 17 H-CCa, and 12 IH-CCa

NR: not reported; H-CCa: hilar cholangiocarcinoma; EH-CCa: extrahepatic cholangiocarcinoma; IH-CCa: intrahepatic cholangiocarcinoma.

**Table 2:** Technical characteristics of the included studies.

Authors	Year	Device	^ 18^F-FDG mean injected dose (MBq)	Time between ^18^F-FDG injection and image acquisition (min)	Image analysis	Other imaging methods performer
Fritscher-Ravens et al. [6]	2001	PET	Range: 320–400	60	Visual and semiquantitative	CT and ERCP

Kluge et al. [7]	2001	PET	370	50	Visual and semiquantitative	US, CT, MR, and ERCP

Kato et al. [8]	2002	PET	NR	60	Visual and semiquantitative	CT

Kim et al. [9]	2003	PET	370	60	Visual and semiquantitative	CR, MR, ERCP, and PTC

Anderson et al. [10]	2004	PET	370	60	Visual	CT and MR

Reinhardt et al. [11]	2005	PET/CT	369	101	Visual and semiquantitative	ERCP

Wakabayashi et al. [12]	2005	PET	185	60	Visual	CT, ERCP, and PTC

Petrowsky et al. [13]	2006	PET/CT	370	45	Visual and semiquantitative	CT, ERCP, and PTC

Prytz et al. [14]	2006	PET	300	Dynamic 0–90	Visual and quantitative	US, CT, MR, ERCP, and PTC

Nishiyama et al. [15]	2007	PET	3/kg	70	Visual and semiquantitative	US, CT, and MR

Corvera et al. [16]	2008	PET	Range: 370–555	NR	Visual and semiquantitative	US, CT, and MR

Furukawa et al. [17]	2008	PET	Range: 200–250	60	Visual	US and CT

Kim et al. [18]	2008	PET/CT	370	60	Visual and semiquantitative	CR, CT, MR, ERCP, and PTC

Li et al. [19]	2008	PET/CT	350	60	Visual and semiquantitative	CT, MR, and ERCP

Moon et al. [20]	2008	PET	370	60	Visual and semiquantitative	CT

Lee et al. [21]	2010	PET/CT	Range: 370–555	60	Visual and semiquantitative	CT and ERCP

Alkhawaldeh et al. [22]	2011	PET/CT	2.52/kg	100	Visual and semiquantitative	ERCP

Kitamura et al. [23]	2011	PET/CT	250	60	Visual and semiquantitative	US, CT, MR, ERCP, PTC, and HS

Ruys et al. [24]	2011	PET	296	50	Visual and semiquantitative	CT, MR, ERCP, and PTC

Yamada et al. [25]	2012	PET	4.5/kg	60	Visual and semiquantitative	CT, MR, and ERCP

Albazaz et al. [3]	2013	PET-CT	400	60	Visual and semiquantitative	CT and MR

Choi et al. [1]	2013	PET/CT	Range: 370–555	60	Visual and semiquantitative	US, CT, and MR

Lee et al. [2]	2013	PET/CT	Range: 370–555	60	Visual and semiquantitative	US, CT, MR, and ERCP

NR: not reported; CT: computed tomography; ERCP: endoscopic retrograde cholangiopancreatography; MR: magnetic resonance; US: ultrasonography; PTC: percutaneous transhepatic cholangiography; HS: hepatobiliary scintigraphy.

**Table 3:** Diagnostic accuracy data of ^18^F-FDG-PET or PET/CT on a per patient-based analysis.

Author	Year	Overall				PET				PET/CT				IH-CCa				H-CCa				EH-CCa			
		TP	FP	FN	TN	TP	FP	FN	TN	TP	FP	FN	TN	TP	FP	FN	TN	TP	FP	FN	TN	TP	FP	FN	TN
Fritscher-Ravens et al. [6]	2001	10	2	3	0	10	2	3	0	NR	NR	NR	NR	NR	NR	NR	NR	10	2	3	0	NR	NR	NR	NR

Kluge et al. [7]	2001	24	2	2	26	24	2	2	26	NR	NR	NR	NR	NR	NR	NR	NR	24	2	2	26	NR	NR	NR	NR

Kato et al. [8]	2002	18	0	12	0	18	0	12	0	NR	NR	NR	NR	NR	NR	NR	NR	NR	NR	NR	NR	18	0	12	0

Kim et al. [9]	2003	20	0	1	0	20	0	1	0	NR	NR	NR	NR	11	0	0	0	9	0	1	0	NR	NR	NR	NR

Anderson et al. [10]	2004	19	1	12	4	19	1	12	4	NR	NR	NR	NR	NR	NR	NR	NR	NR	NR	NR	NR	NR	NR	NR	NR

Reinhardt et al. [11]	2005	19	2	2	7	19	2	2	7	NR	NR	NR	NR	2	0	0	3	NR	NR	NR	NR	12	2	1	2

Wakabayashi et al. [12]	2005	12	0	0	8	NR	NR	NR	NR	12	0	0	8	NR	NR	NR	NR	12	0	0	8	NR	NR	NR	NR

Petrowsky et al. [13]	2006	3	1	1	19	3	1	1	19	NR	NR	NR	NR	NR	NR	NR	NR	NR	NR	NR	NR	NR	NR	NR	NR

Prytz et al. [14]	2006	31	3	16	5	NR	NR	NR	NR	31	3	16	5	13	1	1	4	NR	NR	NR	NR	18	2	15	1

Nishiyama et al. [15]	2007	25	1	4	7	25	1	4	7	NR	NR	NR	NR	NR	NR	NR	NR	NR	NR	NR	NR	25	1	4	7

Corvera et al. [16]	2008	46	1	13	2	46	1	13	2	NR	NR	NR	NR	19	0	1	1	NR	NR	NR	NR	27	1	12	2

Furukawa et al. [17]	2008	40	0	7	0	40	0	7	0	NR	NR	NR	NR	NR	NR	NR	NR	NR	NR	NR	NR	40	0	7	0

Kim et al. [18]	2008	79	6	15	23	NR	NR	NR	NR	79	6	15	23	20	3	1	12	NR	NR	NR	NR	59	3	14	11

Li et al. [19]	2008	41	1	5	7	41	1	5	7	NR	NR	NR	NR	21	1	2	4	10	0	2	1	10	0	1	2

Moon et al. [20]	2008	10	0	7	0	NR	NR	NR	NR	10	0	7	0	NR	NR	NR	NR	10	0	7	0	NR	NR	NR	NR

Lee et al. [21]	2010	69	5	13	12	NR	NR	NR	NR	69	5	13	12	17	0	0	0	NR	NR	NR	NR	38	0	11	0

Alkhawaldeh et al. [22]	2011	45	6	2	12	NR	NR	NR	NR	45	6	2	12	NR	NR	NR	NR	NR	NR	NR	NR	NR	NR	NR	NR

Kitamura et al. [23]	2011	23	4	3	0	NR	NR	NR	NR	23	4	3	0	NR	NR	NR	NR	23	4	3	0	NR	NR	NR	NR

Ruys et al. [24]	2011	50	0	23	0	50	0	23	0	NR	NR	NR	NR	NR	NR	NR	NR	NR	NR	NR	NR	NR	NR	NR	NR

Yamada et al. [25]	2012	47	4	7	0	47	4	7	0	NR	NR	NR	NR	16	3	0	0	14	0	4	0	17	1	3	0

Albazaz et al. [3]	2013	48	1	14	0	NR	NR	NR	NR	48	1	14	0	36	0	3	0	NR	NR	NR	NR	12	1	11	0

Choi et al. [1]	2013	27	1	7	4	NR	NR	NR	NR	27	1	7	4	NR	NR	NR	NR	NR	NR	NR	NR	27	1	7	4

Lee et al. [2]	2013	51	0	10	0	NR	NR	NR	NR	51	0	10	0	NR	NR	NR	NR	NR	NR	NR	NR	NR	NR	NR	NR

NR: not reported; IH-CCa: intrahepatic cholangiocarcinoma; H-CCa: hilar cholangiocarcinoma; EH-CCa: extrahepatic cholangiocarcinoma; TP: true positive; FP: false positive; FN: false negative; TN: true negative.

**Table 4:** Quality assessment of the included studies.

First author/year	Spectrum of patients	Consecutive or random selection of patients	Reference standard	Application of reference standard regardless of indexed test	Enough explanation of the index test to ensure reproducibility	Independent blind comparison between index test and reference standard
Fritscher-Ravens, 2001 [6]	Patients with obstructive jaundice and hilar lesions	Yes	Histopathology	Yes	Yes	Yes

Kluge, 2001 [7]	26 patients with CCa, 8 patients with benign bile duct stenosis, and 20 controls	No	Histopathology	Yes	Yes	Yes

Kato, 2002 [8]	Patients with bile duct cancer	N/A	Histopathology	Yes	Yes	Yes

Kim, 2003 [9]	Patients with intrahepatic CCa	N/A	Histopathology or clinical and radiological findings	Yes	Yes	No blinding

Anderson, 2004 [10]	Patients suspected of CCa or gallbladder carcinoma	Yes	Histopathology or cytopathology	Yes	Yes	No information regarding blinding

Reinhardt, 2005 [11]	Patients with extrahepatic bile duct strictures on endoscopic retrograde cholangiography	Yes	Histopathology or cytopathology and imaging criteria and follow-up	No (patients with negative PET and cytology did not undergo surgery)	Yes	No

Wakabayashi, 2005 [12]	Patients with suspicious malignant biliary stricture	N/A	Histopathology (biopsy and surgical findings)	Yes	Yes	N/A

Petrowsky, 2006 [13]	Patients with suspected or proven CCa or gallbladder cancer	Yes	Histopathology	Yes	Yes	Yes

Prytz, 2006 [14]	Patients with primary sclerosing cholangitis within 2 weeks after listing for liver transplantation and with no evidence of malignancy on CT, magnetic resonance imaging, or ultrasonography	Yes	Histology of explanted livers	Yes	Yes	Yes

Nishiyama, 2007 [15]	Patients with biliary stricture who underwent PET imaging	Yes	Histopathology or cytology	Yes	Yes	Yes

Corvera, 2008 [16]	Patients with clinical diagnosis of biliary tract cancers	N/A	Histopathology	Yes	Yes	No

Furukawa, 2008 [17]	Patients with suspected extrahepatic biliary cancers (bile duct dilatation and/or mass lesions detected by ultrasonography and CT)	N/A	Histopathology and follow-up	Yes	Yes	No (no blinding)

Kim, 2008 [18]	Patients with suspected CCa	Yes	Histopathology or follow-up	Yes	Yes	Yes

Li, 2008 [19]	Patients with clinically suspected or already established diagnosis of hilar CCa	N/A	Histopathology	Yes	Yes	N/A

Moon, 2008 [20]	Patients with suspected CCa	Yes	Histopathology, cytopathology, or follow-up	Yes	Yes	Yes

Lee, 2010 [21]	Patients with suspected CCa or gallbladder cancer	N/A	Histopathology or follow-up	Yes	Yes	Yes

Alkhawaldeh, 2011 [22]	Heterogeneous patients including patients with suspected CCa, patients with positive cytology for CCa, and patients with negative cytology	No	Histopathology or follow-up	Yes	Yes	N/A

Kitamura, 2011 [23]	Patients with extrahepatic bile duct cancer	Yes	Histopathology	Yes	Yes	No

Ruys, 2011 [24]	Patients highly suspected of hilar CCa	Yes	Histopathology	Yes	Yes	Yes

Yamada, 2012 [25]	Patients with diagnosis of cancer of biliary tract	Yes	Histopathology	Yes	Yes	No

Albazaz, 2013 [3]	Patients with primary biliary tumors	Yes	Histopathology	Yes	Yes	Yes

Choi, 2013 [1]	Patients with suspected extrahepatic malignancy based on imaging studies	Yes	Histopathology or follow-up	Yes	Yes	Yes

Lee, 2013 [2]	Patients with confirmed biliary duct or gallbladder cancers	N/A	Histopathology or follow-up	Yes	Yes	N/A

CCa: cholangiocarcinoma.
